# Function of the Ciliopathy gene RPGRIP1L in cortical neurogenesis

**DOI:** 10.1186/2046-2530-4-S1-P83

**Published:** 2015-07-13

**Authors:** G Pézeron, I Anselme, M Catala, C Laclef, S Schneider-Maunoury

**Affiliations:** 1 CNRS UMR7622, Inserm U1156, Sorbonne Universités- UPMC Paris 06, Developmental Biology Laboratory, Paris, France

## Objective

The aim of this work is to identify the functions of the ciliary gene *Rpgrip1l/Ftm *in neurogenesis in the cerebral cortex. *Rpgrip1l/Ftm *encodes a protein enriched at the ciliary transition zone and is involved in ciliopathies with associated brain abnormalities, Meckel and Joubert syndromes. We have previously shown that *Rpgrip1l *is required for telencephalic morphogenesis.

## Methods

We use a mouse knock-out mutant line, *Ftm^KO^*, to identify the role of Rpgrip1l in cortical neurogenesis. Two distinct pools of progenitors undergo extensive cell divisions to form cortical projection neurons. Radial Glial Cells (RGCs) divide either symmetrically to expand the progenitor pool or asymmetrically to self-renew and produce an Intermediate Progenitor Cell (IPC), which divides once to form two neurons. The balance between differentiation and proliferation is coordinated by multiple signalling pathways, among which several depend on the primary cilium.

## Results

In *Ftm *mutant embryos, the cortex is thinner with a reduction in the number of neurons and of IPCs. In contrast, the RGCs are present in normal numbers and they proliferate normally. This suggests a defect in the balance between symmetric (proliferative) and asymmetric (neurogenic) divisions, a hypothesis we are currently confirming. We showed that the reintroduction of a short, repressor form of the Gli3 transcription factor partially rescues cortical neurogenesis in *rpgrip1l *mutant background. We are currently investigating the molecular mechanisms of this defect downstream of Rpgrip1l and Gli3R.

## Conclusion

Our results show that Rpgrip1l controls neurogenesis in the cerebral cortex, via the formation of the Gli3 repressor.

